# Detection of *Wolbachia* and different trypanosome species in *Glossina palpalis palpali*s populations from three sleeping sickness foci of southern Cameroon

**DOI:** 10.1186/s13071-018-3229-2

**Published:** 2018-12-12

**Authors:** Sartrien Tagueu Kanté, Trésor Melachio, Elvis Ofon, Flobert Njiokou, Gustave Simo

**Affiliations:** 1 Molecular Parasitology and Entomology Unit (MPEU), Department of Biochemistry, Faculty of Science, University of Dschang, PO Box 67, Dschang, Cameroon; 2 Laboratory of Parasitology and Ecology, Faculty of Science, University of Yaoundé I, Yaoundé, Cameroon

**Keywords:** *Glossina palpalis palpalis*, Symbiont, *Wolbachia*, Trypanosoma sp

## Abstract

**Background:**

African trypanosomiases are caused by trypanosomes that are cyclically transmitted by tsetse. Investigations aiming to generate knowledge on the bacterial fauna of tsetse have revealed distinct symbiotic microorganisms. Furthermore, studies addressing the tripartite association between trypanosomes-tsetse-symbionts relationship have so far been contradictory. Most studies included *Sodalis glossinudius* and, consequently, the association involving *Wolbachia* is poorly understood. Understanding the vectorial competence of tsetse requires decrypting these tripartite associations. In this study, we identified *Wolbachia* and trypanosomes in *Glossina palpalis palpalis* from three human African trypanosomiasis (HAT) foci in southern Cameroon.

**Methods:**

Tsetse flies were captured with pyramidal traps in the Bipindi, Campo and Fontem HAT foci. After morphological identification, DNA was extracted from whole tsetse flies and *Wolbachia* and trypanosomes were identified by PCR using different trypanosome-specific primers and two *Wolbachia-*specific primers (*Wolbachia* surface protein and *16S* rRNA genes). Statistical analyses were performed to compare the trypanosome and *Wolbachia* infection rates between villages and different foci and to look for an association between these microorganisms.

**Results:**

From a total of 2122 tsetse flies, 790 *G. p. palpalis* were analyzed. About 25.32% of flies hosted *Wolbachia* and 31.84% of non-teneral flies were infected by at least one trypanosome species. There was no significant difference between the global *Wolbachia* prevalence revealed by the two markers while some differences were observed between HAT foci. From 248 *G. p. palpalis* with trypanosome infections, 62.90% were with *T. vivax*, 34.68% with *T. congolense* forest, 16.13% with *T. brucei* (*s.l.*) and 2.42% with *T. congolense* savannah. Of all trypanosome-infected flies, 29.84% hosted *Wolbachia* and no association was observed between *Wolbachia* and trypanosome co-infections.

**Conclusions:**

This study revealed differences in the prevalence of *Wolbachia* and trypanosomes in *G. p. palpalis* according to HAT foci*.* The use of only one marker has underestimated the prevalence of *Wolbachia*, thus more markers in subsequent studies may improve its detection. The presence of *Wolbachia* seems to have no impact on the establishment of trypanosomes in *G. p. palpalis*. The tripartite association between tsetse, *Wolbachia* and trypanosomes varies according to studied areas. Studies aiming to evaluate the genetic polymorphism of *Wolbachia* and its density in tsetse flies could help to better understand this association.

## Background

Tsetse flies are dipteran insects of the genus *Glossina.* With a certain number of requirements linked to environmental factors such as the climate, the vegetation, the type of soil, the presence of domestic and/or wild fauna, and the effects of human activity, the distribution of tsetse flies is discontinuous across 37 sub-Saharan countries. Tsetse flies are the cyclical vector of African trypanosomes that cause human and animal African trypanosomiases. Two subspecies of African trypanosomes are pathogenic for humans: *Trypanosoma brucei rhodesiense* that causes the acute form of human African trypanosomiasis (HAT) in eastern and southern Africa, and *T. b. gambiense* which is responsible for the chronic form of HAT in western and central Africa [1, 2]. About 60 million people are exposed to the risk of HAT and, for the first time in 2016 and up to date, the number of reported cases is below 3000 [3, 4]. In recent decades, efforts undertaken on HAT control have brought the disease under control and led to its inclusion into the WHO “roadmap for elimination of neglected tropical diseases” with a target set to eliminate HAT as a public health problem by 2020 [1].

Alongside *T. b. rhodesiense* and *T. b. gambiense*, other African trypanosomes including *T. b. brucei*, *T. congolense*, *T. vivax* and *T. simiae* are responsible for the animal African trypanosomiasis (AAT) or “nagana” in animals. AAT is one of the biggest constraints to livestock production and a threat to food security in sub-Saharan Africa. Human and animal trypanosomiases have impacts on human and animal health, but also on animal productivity and, therefore, the peasant economy. In both human and animal trypanosomiases, tsetse flies play a key role in the transmission of parasites between different vertebrate hosts. To achieve HAT elimination and boost AAT control, the integration of vector control as component of new control strategies is becoming crucial. A better understanding of how trypanosomes develop in tsetse flies appears to be an important step in the process leading to the development of innovative vector control strategies. In recent decades, growing interests have been focused on tripartite interactions between trypanosomes, tsetse fly and tsetse-associated symbiotic microorganisms. Currently, three symbiotic microorganisms including *Wigglesworthia glossinidia*, *Sodalis glossinidius* and *Wolbachia* have been reported to be associated with tsetse flies. While *W. glossinidia* is an obligate primary symbiont, *S. glossinidius* is a secondary and a non-essential symbiont which seems to affect vector competence of tsetse by favoring the midgut establishment of trypanosomes through a complex biochemical mechanism. *Wolbachia* spp. are also non-essential symbionts that infect a wide range of invertebrates. Abundant in both male and female germ-cells and also in the somatic tissues, *Wolbachia* spp. are found in a wide range of arthropods [5] and nematodes [6]. Transmitted vertically from mother to offspring [7], *Wolbachia* can protect their hosts against viral pathogens [8]. It has the ability to induce cytoplasmic incompatibility that leads to embryonic death in tsetse flies [9, 10]. Investigations on *Wolbachia* in tsetse populations may improve vector control through the development of transgenic tsetse with the ability to release specific molecules that can interfere with the establishment of trypanosomes. Previous studies reported *Wolbachia* in several tsetse species from insectariums and few wild tsetse populations such as *Glossina morsitans morsitans*, *G. m. centralis*, *G. f. fuscipes*, *G. austeni*, *G. pallidipes* and *G. brevipalpis* [11–15]. Investigations on the tripartite association between trypanosomes, tsetse fly and its symbiotic microorganisms reported contrasting results. For instance, Alam et al. [14] reported a negative association between *Wolbachia* and trypanosome infections in *G. f. fuscipes*, suggesting that the presence of *Wolbachia* could prevent trypanosome infections. Despite these interesting results, little investigation has been undertaken on the tripartite association between tsetse fly, trypanosomes and *Wolbachia*, and therefore this tripartite association is not well understood. A better understanding of this association requires the collection of more data on trypanosome and symbiont infections in different tsetse species from various tsetse infested areas. In tsetse flies of the *palpalis* group, investigations on the tripartite association were focused essentially on *S. glossinidius* and trypanosomes [16, 17]. These investigations revealed a positive association between the presence of *S. glossinidius* and trypanosome infections [16, 17]. However, there is currently very little, if any, data on the tripartite association involving *Wolbachia* in tsetse of the *palpalis* group.

In the present study, *Wolbachia* and different trypanosome species were identified in wild populations of *G. p. palpalis* caught in three sleeping sickness foci of southern Cameroon with the final goal of generating data that may shed more light on the tripartite association and help to understand the impact of *Wolbachia* infections on the transmission of African trypanosomes.

## Methods

### Study zones

**Fig. 1:**
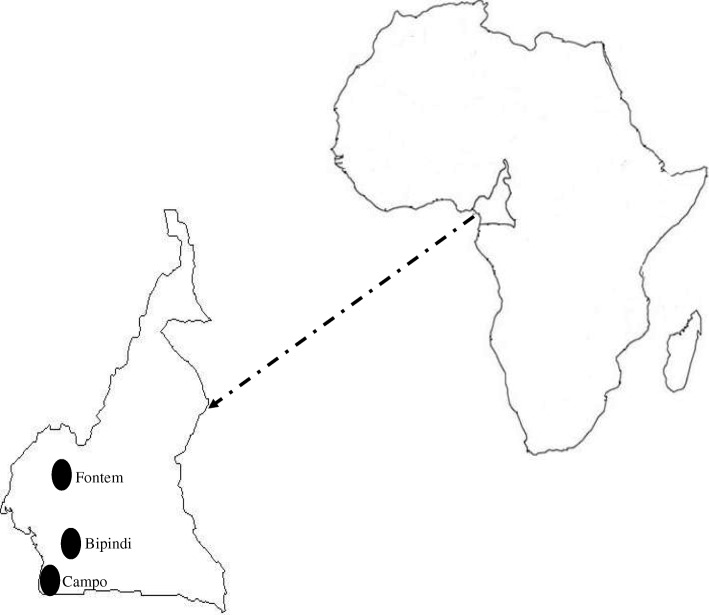
Map showing sleeping sickness foci where tsetse flies were caught (circles)

This study was performed in the Bipindi, Campo and Fontem HAT foci located in the forest region of southern Cameroon (Fig. 1). The Bipindi and Campo HAT foci are located in the Ocean Division of the South Region of Cameroon. The Campo HAT focus offers several types of biotopes (farmland, swampy areas and equatorial forest) while the Bipindi HAT focus shows a typical forest bioecological environment.

The Bipindi (3°2'00"N, 10°22'00"E) HAT focus has been known since 1920 [18]. It covers several villages located along the roads and its bio-ecological environment is characterized typically by an equatorial forest with farmland along the roads and the villages. The wild fauna composition is highly diversified [19]. Peasant agriculture, hunting, fishing and breeding of livestock are the main socioeconomic activities. The focus is surrounded by hills and has a dense hydrographic network with many rivers crossing farmlands. The bioclimatic environment offers suitable habitats for tsetse flies.

The Campo HAT focus (2°22'00"N, 9°49'00"E) lies along the Atlantic coast and extends along the Ntem River which constitutes the border of Cameroon and Equatorial Guinea. This focus is characterized by an equatorial rainforest with a hydrographic network containing several rivers and swampy areas. Its fauna composition is highly diversified. The climate is typical maritime equatorial comprising four seasons. The main activities of the inhabitants are fishing, picking fruits, hunting and farming.

The Fontem HAT focus (5°40'12"N, 9°55'33"E) is located in the Lebialem division of the southwest region of Cameroon. It is characterized by a tropical humid climate, having an irregular relief with many hills and valleys that are crossed by fast-flowing streams. The main population activities are subsistence agriculture, palm oil extraction, animal husbandry and small-scale poultry farming. The dense population of humans, domestic animals (dogs, pigs, sheep and goats) and tsetse flies are found scattered in the pre-forest/forest vegetation of the valleys and hills.

### Trapping of tsetse flies

Tsetse flies were collected during four entomological surveys in the three HAT foci of Cameroon. During the first survey in the Campo HAT focus in 2012, tsetse flies were trapped at Akak, Campo beach, Ipono and Mabiogo villages. During the second survey in 2015, tsetse flies were trapped at Bidjouka, Ebiminbang and Lambi villages of the Bipindi HAT focus. The third and fourth surveys were performed in 2015 and 2017 at Bechati, Besali Folepi and Menji villages in the Fontem HAT focus. During each survey, pyramidal traps [20] were set for 4 consecutive days. In total, 197 traps were set up: 105 at Campo, 50 at Bipindi and 42 at Fontem. The geographical coordinates of each trap were recorded using a global positioning system (GPS). Tsetse flies were collected twice a day. All collected flies were morphologically identified, counted and sorted into teneral and non-teneral flies as described by Pollock [21]. Thereafter, each identified fly was put into a microtube containing 95% ethanol. In the field, microtubes were kept at room temperature, and in the laboratory they were stored at -20 °C.

### DNA extraction

DNA was extracted from whole tsetse fly using the cetyl trimethyl ammonium bromide (CTAB) method as described by Navajas et al. [22]. Briefly, the alcohol preserving each fly was evaporated by incubating the opened microtubes at 80 °C in an oven for about 1 h. Thereafter, each tsetse fly was disrupted with a pestle in CTAB buffer (CTAB 2%; 1 M Tris, pH 8; 0.5 M EDTA pH 8; 5 M NaCl). The disrupted fly was incubated at 60 °C for 30 min before the addition of chloroform/isoamylic alcohol mixture (24/1, V/V). DNA was precipitated by addition of isopropanol (V/V) followed by centrifugation at 13,000× *rpm* for 15 min. The resulting DNA pellets were washed twice with cold 70% ethanol and then dried overnight at room temperature. DNA pellets were finally re-suspended in 50 µl of sterile water before storing at -20 °C until use.

### Molecular identification of *Wolbachia*

The identification of *Wolbachia* was performed using two sets of primers. The first set of primers, wspec F1 (5'-YAT ACC TAT TCG AAG GGA TAG-3') and wspec R1 (5'-AGC TTC GAG TGA AAC CAA TTC-3'), described by Werren & Windsor [23], amplifies a fragment of the *16S* rRNA gene. The second set of primers, wsp F_1_ (5'-GTC CAA TAR STG ATG ARG AAA C-3') and wsp R_1_ (5'-CYG CAC CAA YAG YRC TRT AAA-3'), described by Baldo et al. [24], amplifies a fragment of the *Wolbachia* surface protein gene. All PCR reactions were performed in a final volume of 15 μl containing 3 μl of DNA extract, 1.5 μl of 10× PCR reaction buffer, 2 mM MgCl_2_, 20 pmol of each primer, 200 mM of each dNTP and 0.3 units of Taq DNA polymerase (New England Biolabs, Massachusetts, USA; 5U/μl). The amplification program comprised an initial denaturation step at 94 °C for 3 min followed by 37 amplification cycles of denaturation at 94 °C for 30 s, annealing at 54 °C (wspec) or 53 °C (wsp) for 30 s, and extension at 72 °C for 1 min. A final extension was performed at 72 °C for 5 min.

At the end of PCR reactions, 10 μl of amplified product was analyzed by electrophoresis on 2% agarose gel containing ethidium bromide. Each gel was visualized under UV light and then photographed.

### Detection of trypanosomes

Different trypanosome species including *T. brucei* (*s.l.*), *T. vivax*, *T. congolense* forest type and *T. congolense* savannah type were investigated. Trypanosome identification was performed as previously described by Herder et al. [25] using the primers TCF1/2 (5'-GGA CAC GCC AGA AGG TAC TT-3'; 5'-GTT CTC GCA CCA AAT CCA AC-3') for *T. congolense* forest type [26], TCN1/2 (5'-TCG AGC GAG AAC GGG CAC TTT GCG A-3'; 5'-ATT AGG GAC AAA CAA ATC CCG CAC A-3') for *T. congolense* savannah type [27], TBR1/2 (5'-CGA ATG AAT ATT AAA CAA TGC GCA G-3'; 5'-AGA ACC ATT TAT TAG CTT TGT TGC-3') for *T. brucei* (*s.l.*) [26] and TVW1/2 (5'-CTG AGT GCT CCA TGT CCC AC-3'; 5'-CCA CCA GAA CAC CAA CCT GA-3') for *T. vivax* [26]. The amplification reaction was carried out in a final volume of 15 μl containing 1.5 μl of 10× PCR reaction buffer, 1.5 mM MgCl_2_, 0.5 μl of dNTPs (200 mM for each dNTP), 1 μl (10 pmol) of each primer, 0.3 U of Taq DNA polymerase (New England Biolabs; 5U/μl), 3 μl of DNA extract, and nuclease-free water. The amplification program comprised an initial denaturation step at 94 °C for 5 min, followed by 40 amplification cycles of denaturation at 94 °C for 30 s, annealing at 60 °C for 30 s for the four trypanosome species investigated in this study, and an extension step at 72 °C for 1 min. A final extension was performed at 72 °C for 10 min.

Amplified products were resolved on 2% agarose gel containing ethidium bromide and visualized under UV light.

### Statistical analysis

Statistical analyses were performed using the R 3.4.1 [28]. A Chi-square test was used to compare, between foci, the infection rates of *Wolbachia* sp. and different trypanosome species. The differences were considered significant when *P*-values were lower than 0.05. To analyze the relationship between *Wolbachia* sp. and trypanosome infections, a generalized linear model (package *stats* in R) was used with 95% confidence intervals (CIs). *Trypanosoma vivax* was excluded for these analyses because its life-cycle is restricted to the mouthparts of tsetse flies.

## Results

### Entomological surveys

From the 197 traps used in this study, a total of 2122 tsetse flies were collected during the four entomological surveys: 1216 (57.3%) tsetse flies were caught in the Bipindi HAT focus, 632 (29.78%) in the Campo focus and 274 (12.91%) in the Fontem focus. Four different tsetse species and subspecies including *G. caliginea*, *G. tabaniformis*, *G. p. palpalis* and *G. p. pallicera* were identified. *Glossina p. palpalis* was the only tsetse subspecies caught in the Fontem HAT focus. In the Campo HAT focus, 632 tsetse flies were identified, of which 619 (97.94%) were *G. p. palpalis*, 9 (1.42%) *G. pallicera*, 3 (0.47%) *G. tabaniformis* and 1 (0.16%) *G. caliginea*. In the Bipindi HAT focus, 1216 tsetse flies were identified, of which 1208 (99.34%) were *G. p. palpalis* and 8 (0.66%) *G. pallicera*. In the three HAT foci, 34 (1.6%) teneral flies were identified: 1 (0.05%) at Bipindi, 24 (1.13%) at Campo and 9 (0.42%) at Fontem. For the molecular identification of *Wolbachia* and different trypanosome species, 790 (37.23%) *G. p. palpalis* were randomly selected.

### Molecular identification of *Wolbachia*

From 790 tsetse flies that were randomly selected and analyzed by two set of primers (wspec F_1_/wspec R_1_ and wsp F_1_/wsp R_1_), at least one of the two markers identified *Wolbachia* infections in a total of 200 tsetse flies. This gave a global infection rate of 25.32% (200/790) (Table 1).

**Table 1:** Infection rates of *Wolbachia* according to villages and different HAT foci

Focus	Village	No. of flies captured	No. of flies analyzed	No. of flies hosting *Wolbachia* (%)	95% CI
Bipindi	Bidjouka	600	77	17 (22.08)	14.27–32.54
	Ebimimbang	122	46	14 (30.43)	19.08–44.81
	Lambi	486	86	23 (26.74)	18.53–36.95
	Total (1)	1208	209	54 (25.84)	
	*P*-value			0.5734	
Campo	Akak	212	142	29 (20.42)	14.61–27.79
	Campo beach	157	27	12 (44.44)	27.59–62.69
	Ipono	72	72	12 (16.67)	9.8–26.91
	Mabiogo	178	66	9 (13.64)	7.34–23.93
	Total (2)	619	307	62 (20.2)	
	*P*-value			0.0068	
Fontem	Bechati	54	54	9 (16.67)	9.02–28.74
	Besali	4	4	1 (25)	
	Folepi	145	145	23 (15.86)	10.81–22.68
	Menji	71	71	51 (71.83)	60.46–80.96
	Total (3)	274	274	84 (30.66)	
	*P-* value			< 2.2e-16	
Total (1) + (2) + (3)		2101	790	200 (25.32)	
*P*-value				0.0309	

*Abbreviations*: *% Wolbachia* infection rate, *CI* confidence interval

The highest infection rate of 71.83% was observed in tsetse flies caught at Menji in the Fontem HAT focus and the lowest infection rate of 13.64% in flies caught at Mabiogo in the Campo HAT focus. Between HAT foci, a significant difference (*χ*^2^ = 6.9543, *df* = 2, *P* = 0.0309) was observed in the *Wolbachia* infection rates. Similar results were observed between villages of the same HAT focus, except in the Bipindi focus where the difference in the *Wolbachia* infection rates was not significant (*χ*^2^ = 1.1123, *df* = 2, *P* = 0.5734) (Table 1)*.*

### Comparison of results generated by *16S* rDNA and WSP markers

Of the 200 tsetse flies with *Wolbachia* infections, 130 (65%) were positive for the *16S* DNA marker and 121 (60.5%) for WSP. Fifty-one (25.5%; 51/200) of these infections were simultaneously identified by both *16S* and WSP markers (Table 2). However, no significant difference (*χ*^2^ = 0.3837, *df* = 1, *P* = 0.5357) was observed between the number of *Wolbachia* infections identified by these two markers.

**Table 2:** *Wolbachia* infections according to different HAT foci

HAT foci	No. of samples analysed	*Wolbachia* infections						
		*16S* rDNA (%)	95% CI	WSP (%)	95% CI	*P-*value	*16S* rDNA and WSP (%)	95% CI
Bipindi	209	26 (12.4)	8.6–17.6	40 (19.1)	14.4–25.0	0.0604	12 (5.7)	3.3–9.8
Campo	307	29 (9.5)	6.7–13.2	62 (20.2)	16.1–25.0	0.0002	29 (9.5)	6.7–13.2
Fontem	274	75 (27.4)	22.4–32.9	19 (6.9)	4.5–10.6	2.213e-10	10 (3.7)	209–6.6
Total	790	130 (16.5)		121 (15.3)		0.5357	51 (6.5)	
*P-*value		8.466e-09		0.00001			0.0158	

*Abbreviations*: *%*, *Wolbachia* infection rate, *CI* confidence interval

In the Bipindi and Campo HAT foci, WSP appeared more sensitive because 40 (19.14%) and 62 (20.19%) *Wolbachia* infections, respectively, were identified by this marker while only 26 (12.44%) and 29 (9.45%) infections, respectively, were identified by *16S* in the same focus. *Wolbachia* infections simultaneously identified by the two markers in the Bipindi and Campo HAT foci were 12 (5.74%; 12/209) and 29 (9.45%; 29/307), respectively. The difference in sensitivity between these markers was significant in the Campo (*χ*^2^ = 14.049, *df* = 1, *P* = 0.0002) HAT focus, but not in the Bipindi (*χ*^2^ = 3.5265, *df* = 1, *P* = 0.0604) HAT focus (Table 2).

In the Fontem HAT focus, *16S* rDNA detected significantly (*χ*^2^ = 40.269, *df* = 1, *P* < 0.0001) more infections than WSP (Table 2): 75 (27.37%) *Wolbachia* infections were identified by *16S* compared to 19 (6.93%) identified by WSP. In this focus, only 3.65% (10/274) of *Wolbachia* infections were simultaneously detected by *16S* and WSP (Table 2).

Regardless of the marker used in this study (*16S* or WSP), a significant difference (*χ*^2^ = 22.831, *df* = 2, *P* <0.0001 for WSP; *χ*^2^ = 8.296, *df* = 2, *P* = 0.0158 for *16S* rDNA) was observed in the *Wolbachia* infection rates between all the three HAT foci (Table 2).

### Molecular detection of different trypanosome species

From 790 tsetse flies randomly selected, 11 were teneral flies. These 11 flies were excluded from the identification of trypanosomes because they had never taken a blood meal. From the remaining 779 non-teneral flies that were subjected to molecular identification of trypanosomes, 248 (31.84%) were infected with at least one trypanosome species: 156 (62.90%) with *T. vivax*; 86 (34.68%) with *T. congolense* forest type; 40 (16.13%) *T. brucei* (*s.l.*) and 6 (2.42%) *T. congolense* savannah type (Table 3). Between villages of the same HAT focus, no significant difference was observed in the infection rates of different trypanosome species except for *T. congolense* forest type (*χ*^2^ = 10.254, *df* = 2, *P* = 0.0059) in the Fontem HAT focus (Table 3).

**Table 3:** Trypanosome infections according to villages and different HAT foci

Focus	Village	No. of flies captured	No. of flies analyzed	Trypanosome infections [95% CI]				
				T+ (%)	Tb (*s.l.*) (%)	Tcf (%)	Tcn (%)	Tv (%)
Bipindi	Bidjouka	600	77	17 (22.1)	0 (0)	0 (0)	0 (0)	17 (22.1)
	Ebimimbang	122	46	9 (19.6)	0 (0)	0 (0)	0 (0)	9 (19.6)
	Lambi	486	86	16 (18.6)	0 (0)	0 (0)	0 (0)	16 (18.6)
	Total (1)	1208	209	42 (20.1) [15.2–26.1]	0 (0)	0 (0)	0 (0)	42 (20.1) [15.2–26.1]
	*P-*value			0.854				0.854
Campo	Akak	212	141	42 (29.8)^a^	14 (9.9)	11 (7.8)	2 (1.4)	28 (19.9)
	Campo beach/center	157	27	9 (33.3)^a^	2 (7.4)	1 (3.7)	0 (0)	7 (25.9)
	Ipono	72	71	18 (25.4)^a^	0 (0)	4 (5.6)	4 (5.6)	13 (18.3)
	Mabiogo	178	66	37 (56.1)^a^	24 (36.4)	1 (1.5)	0 (0)	18 (27.3)
	Total (2)	619	305	106 (34.8) [29.6–40.3]^a^	40 (13.1) [9.8–17.4]	17 (5.6) [3.51–8.74]	6 (2.0) [0.9–4.2]	66 (21.6) [17.4–26.6]
	*P-*value			0.0005				0.521
Fontem	Bechati	54	53	18 (34.0)^a^	0 (0)	9 (16.98)	0 (0)	11 (20.8)
	Besali	4	4	1 (25)^a^	0 (0)	1 (25)	0 (0)	1 (25)
	Folepi	145	138	48 (34.9)^a^	0 (0)	31 (22.46)	0 (0)	22 (15.9)
	Menji	71	70	33 (47.1)^a^	0 (0)	28 (40)	0 (0)	14 (20)
	Total (3)	274	265	100 (37.7) [32.1–43.7]^a^	0 (0)	69 (26.04) [21.1–31.6]	0 (0)	48 (18.1) [13.9–23.2]
	*P-*value			0.1774		0.0059		0.6512
Total (1) + (2) + (3)		2101	779	248 (31.8) [28.7–35.2]^a^	40 (5.1) [3.8–6.9]	86 (11.0) [9.0–13.4]	6 (0.8) [0.35–1.7]	156 (20.1) [17.4–23.0]
*P-*value				7.76e-05				0.5596

^a^Parts of tsetse flies with co-infections of different trypanosome species

*Abbreviations*: *%* trypanosome infection rate, *T+* tsetse flies with at least one trypanosome infection, *Tb (s.l.) Trypanosoma brucei sensu lato*, *Tcf Trypanosoma congolense* forest type, *Tcn Trypanosoma congolense* savannah type, *Tv Trypanosoma vivax*, *CI* confidence interval

*Trypanosoma vivax* was found in tsetse flies caught in the three HAT foci. Its prevalence in tsetse flies was 21.64% in the Campo HAT focus, 20.1% in the Bipindi HAT focus and 18.11% in the Fontem HAT focus. The highest infection rate of 27.27% was observed at Mabiogo in the Campo HAT focus and the lowest infection rate of 15.94% in tsetse captured at Folepi in the Fontem HAT focus (Table 3). Between HAT foci, no significant difference (*χ*^2^ = 1.161, *df* = 2, *P =* 0.5596) was observed in the infection rates of *T. vivax*.

*Trypanosoma congolense* savannah type and *T. brucei* (*s.l.*) were found in tsetse from the Campo HAT focus. Their prevalences were 1.97% for *T. congolense* savannah type and 13.11% for *T. brucei* (*s.l.*). The highest infection rate of *T. congolense* savannah type (5.63%) and *T. brucei* (*s.l.*) (36.36%) were found at Ipono and Mabiogo, respectively, of the Campo HAT focus*.* The lowest infection rates of *T. congolense* savannah type (1.42%) and *T. brucei* (*s.l.*) (7.41%) were found at Akak and Campo beach, respectively.

*Trypanosoma congolense* forest type was identified in tsetse caught in the Campo and Fontem HAT foci. Its prevalence was 5.57% in tsetse of the Campo HAT focus and 26.04% in those of the Fontem HAT focus.

Thirty-five (14.11%; 32/248) co-infections comprising 5 triple and 30 double infections were observed. The double infections included 18 (7.26%) *T. congolense* forest type + *T. vivax*, 8 (3.22%) *T. brucei* (*s.l.*) + *T. vivax*, 2 (0.81%) *T. congolense* forest type + *T. brucei* (*s.l.*), 1 (0.4%) *T. congolense* forest type + *T. congolense* savannah type, and 1 (0.4%) *T. vivax* + *T. congolense* savannah type. The five triple infections were composed of 3 (1.21%) infections with *T. congolense* forest type + *T. vivax* + *T. brucei* (*s.l.*), 1 (0.4%) with *T. congolense* forest type + *T. congolense* savannah type + *T. vivax*, and 1 (0.4%) with *T. congolense* forest type + *T. congolense* savannah type + *T. brucei* (*s.l.*).

### *Wolbachia* and trypanosome co-infection

From tsetse flies that were simultaneously analyzed for the presence of *Wolbachia* and different trypanosome species, 25.4% (198/779) harbored *Wolbachia* and 31.8% (248/779) were infected with at least one trypanosome species. Considering the fact that *T. vivax* is found exclusively in the mouthparts, single infections involving only this parasite were excluded from association studies between *Wolbachia* and trypanosomes. With these criteria, 125 flies with single infection of *T. vivax* were excluded. The 31 flies with double and triple infections involving *T. vivax* were considered for association studies. With the exclusion of 125 flies with single infections of *T. vivax*, only 123 tsetse flies with trypanosome infections were subjected to association studies. The Bipindi HAT focus was also excluded from these investigations because only infections due to *T*. *vivax* were found in tsetse caught in this focus. From 123 flies with trypanosome infections, 37 (30.08%) hosted *Wolbachia* (W+T+) while the remaining 86 (69.92%) were devoid of *Wolbachia* (W-T+) (Table 4). Fifty-six tsetse flies hosting *Wolbachia* were infected by *T. vivax*. These 56 flies were also excluded and consequently only 144 tsetse flies (W+) hosting *Wolbachia* were considered for association studies. No trypanosomes were identified in 38.89% (56/144) (W+T-) of these 144 flies. A total of 340 (59.65%) tsetse flies (W-T-) were devoid of infection with either trypanosome or *Wolbachia* (Table 4).

**Table 4:** Combined results of *Wolbachia* and trypanosome infections according to different HAT foci

Focus	No. of flies analyzed	W+T-	W+T+	W-T+	W-T-	W+	T+	*r*	*P-* value of glm	95% CI
Campo	305	51	11	43	200	62	54	0.009	0.993	-0.768–0.705
Fontem	265	56	26	43	140	82	69	1.403	0.161	-0.171–0.987
Total	570	107	37	86	340	144	123			

*Abbreviations*: *W+* tsetse flies infected with *Wolbachia* sp., *T+* tsetse flies infected with at least one trypanosome species, *W+T+* tsetse flies co-infected with *Wolbachia* sp. and at least one trypanosome species, *W+T-* tsetse flies infected with *Wolbachia* sp. but without trypanosome infection, *W-T+* tsetse flies without *Wolbachia* sp. but infected with at least one trypanosome species, *W-T-* tsetse flies infected with neither *Wolbachia* sp. nor trypanosome species, *CI* confidence interval, *glm* generalized linear model, *r* generalized linear model coefficient

A generalized linear model (glm), used to test whether the presence of *Wolbachia* could have impact on the trypanosome infections, revealed no significant association between the two microorganisms in the Campo HAT focus (*r* = 0.009, *P* = 0.993; 95% CI: -0.77–0.71) and in the Fontem HAT focus (*r* = 1.403, *P* = 0.161; 95% CI: -0.17–0.99) (Table 4).

## Discussion

The entomological surveys revealed four tsetse subspecies, with *G. p. palpalis* being the predominant subspecies in the three HAT foci. These data confirm previous reports and highlight not only the great adaptability of *G. p. palpalis*, but also the fact that it is the main vector of African trypanosomes in southern Cameroon [17, 29–32]. The variation of tsetse subspecies according to HAT foci (only *G. p. palpalis* at Fontem, *G. p. palpalis* and *G. p. pallicera* at Bipindi and four species at Campo) indicates how the bio-ecological and bioclimatic conditions characterizing each HAT focus may have impacts on the tsetse fauna [30–32]. The unusual presence of *G. tabaniformis* suggests either an advance of human activity towards areas where it is usually confined or its incursion into anthropized areas where they can easily find vertebrate hosts for their blood meals.

The identification of *Wolbachia* in *G. p. palpalis* contrasts with results of Cheng et al. [15] and Doudoumis et al. [13] who did not identify *Wolbachia* in *G. p. palpalis* as in other tsetse of the *fuscipes* group. Our results are in line with those of Schneider et al. [33] who used sensitive PCR based-methods and identified *Wolbachia* in *G. f. fuscipes*. The sensitivity of molecular markers and the method used to detect *Wolbachia* are important factors that can affect *Wolbachia* infection rates. In our study where two markers (*16S* rDNA and WSP) were used, neither was more sensitive because no significant difference (*P* = 0.53) was observed between their overall performances. However, between HAT foci, significant differences were observed in the infection rates identified by these markers. For instance, WSP was two-fold more sensitive in the Campo focus (20.19% of infections with WSP against 9.45% for *16S* rDNA) while *16S* rDNA showed higher sensitivity (25.37% against 6.93% for WSP) in the Fontem HAT focus. If one marker had been used, the overall *Wolbachia* prevalence would have been approximately 15.32% for WSP and 16.45% for *16S* rDNA. Each single marker underestimated the *Wolbachia* prevalence since about 9% of infections would have not been detected by each of them. The use of two markers improved the detection of *Wolbachia*. Our results suggest the combination of two markers for accurate identification of *Wolbachia* and the need to develop new bio-makers for reliable detection of *Wolbachia* infections. The identification of *Wolbachia* in natural populations of *G. p. palpalis* has important implications for the development of new strategies for vector control. With its ability to induce cytoplasmic incompatibility and to be transmitted from mother to offspring, *Wolbachia* can be genetically modified in order to produce bio-molecules that can interfere with the establishment and/or development of trypanosomes in tsetse flies. This can affect the vectorial competence of tsetse and disease transmission could be blocked through the genetically modified *Wolbachia* strains that conferred resistance to tsetse fly.

The overall *Wolbachia* infection rate of 25.32% is lower than the 44.3%, 98% and 100% reported in *G. f. fuscipes* [14], *G. austeni* [15] and *G. m. morsitans* [13], respectively. These differences could be related to specific biological characteristics of each tsetse subspecies. Indeed, for identical stimulus, interactions between tsetse and its symbiotic microorganisms vary with biological response of each tsetse subspecies. Such variations affect interactions between tsetse and its symbionts and, consequently, the *Wolbachia* infection rates*.* Variations of analytical methods could also explain these differences. In the present study, whole tsetse was used, while in other studies investigations were performed on isolated tissues. It is also plausible that there is a low density of *Wolbachia* in *G. p. palpalis* as already reported in *Rhagoletis cerasi* [34] and *Drosophila paulistorum* [35]. This hypothesis is strengthened by results of Wamwiri et al. [12] where *G. austeni* populations from Kenya had a high density of *Wolbachia* compared to those of South Africa. This low density could explain results of Doudoumis et al. [13] reporting no *Wolbachia* in *G. p. palpalis*.

The differences in the *Wolbachia* infection rates according to HAT foci are in line with observations reported elsewhere [12, 13, 15]. These differences could be related to eco-climatic conditions characterizing each focus. Although the three HAT foci are all located in the forest region of southern Cameroon, each of them is characterized by specific environmental and bio-climatic conditions that have impacts on tsetse biology, its symbiotic microorganisms and finally on the interactions between tsetse and its symbionts. Between villages of the same HAT focus, the differences observed in the *Wolbachia* infection rates can be linked to specific microclimates encountered in each village. This hypothesis is in line with observations reporting that in habitats where environmental conditions fluctuate slightly, the interaction between tsetse and its symbiotic microorganism is stable as well as the transmission of symbionts from mother to offspring [15]. A better understanding of the vector competence of tsetse requires considering the variability of biotopes within and between tsetse infested regions.

The identification of different trypanosomes confirms previous results [31, 32, 36] and indicates current transmission of these parasites. The co-existence of *T. congolense* forest and savannah types indicates that the geographical limit (*T. congolense* savannah and forest in the savannah and forest zones, respectively) tends to change with time. Between HAT foci, the significant differences observed in the trypanosome infection rates could be explained by the fauna composition and the contact frequency between tsetse and mammals.

Regardless of the HAT focus considered here, an increase was observed when our trypanosome infection rates were compared with those previously generated in the same HAT foci (35.1% against 32.4% at Campo in 2008; 20.09% against 9.8% at Bipindi in 2010 and 37.73% against 6.3% at Fontem in 2006) [17, 31, 37]. These results could be explained by the fact that whole tsetse was investigated in our study while previous investigations were undertaken mostly on tsetse midguts. However, the number of flies with immature, mature infections and mouth part infections are unknown with our approach.

In addition to simple infections, our results showed that approximately 14.11% of tsetse of southern Cameroon carried mixed infections of different trypanosome species. These results are in agreement with those of previous studies reporting mixed infections in animals and different tsetse subspecies of Cameroon [36–38] and other African countries [39–41]. Since whole tsetse was analyzed, no information could be inferred from the part of tsetse that was co-infected by trypanosomes. It is difficult to know the proportion of mature co-infections of different trypanosome species. Remarkably, some trypanosomes of triple infections (*T. congolense* forest type, *T. congolense* savannah type and *T. vivax*) can be found in their mature forms in tsetse mouthparts. This highlights a high probability that several trypanosome species might be transmitted by a tsetse fly during a single blood meal on a vertebrate host. In such context, further investigations are required to understand which parasite will establish in the host and how mixed infections will impact the trypanosome transmission dynamics and animal health. With a high number of double and triple infections, there is a need to understand the evolution of these infections and their potential impacts on the transmission dynamics of trypanosomes.

The high trypanosome infection rates indicate not only their high transmission in the forest regions of southern Cameroon, but also the need to implement and intensify control operations to achieve HAT elimination and reduce the incidence of AAT. The identification of trypanosomes in whole tsetse generated more data on trypanosome infections and highlighted AAT as threat for animal health in HAT foci of the southern Cameroon.

The 29.84% of tsetse with co-infections of *Wolbachia* and trypanosomes corroborates results of Alam et al. [14] and Aksoy et al. [42]. This relatively low co-infection rate can be related to the biological effects that this bacterium has on various parasites [14]. The absence of significant correlation between *Wolbachia* and trypanosome infections (Table 4) suggests that the presence of *Wolbachia* does not seem to be an obstacle for the establishment of trypanosomes. This result contrasts the negative correlation reported in *G. f. fuscipes* by Alam et al. [14] who subsequently suggested the prevention of trypanosome infections by the presence of *Wolbachia*. The tripartite association between tsetse, *Wolbachia* and trypanosomes seems to vary according to tsetse subspecies and tsetse populations. Obtaining an overview of the vector competence of tsetse requires also taking into consideration the teneral status of tsetse and its first blood meal on a non-infected host because these factors affect its ability to be infected and could mitigate the influence of symbiotic microorganisms.

With the differences observed in the sensitivity of markers and the presence of tsetse with *Wolbachia* infections and/or without trypanosomes, additional investigations on these bacteria are needed. Instead of focusing only on the presence/absence of *Wolbachia*, investigations aiming to characterize *Wolbachia* and to determine its density could generate additional data that may help to better understand *Wolbachia* infections as well as the contribution of this bacterium in the vector competence of tsetse flies. Moreover, as *Wolbachia* is maternally transmitted from mother to offspring, studies on population genetics of tsetse coupled with *Wolbachia* identification could enable to understand the differences in the susceptibility of different tsetse genotypes to *Wolbachia* infections.

## Conclusions

The present study reveals significant differences in the infections rates of *Wolbachia* and trypanosomes in *G. p. palpalis* from HAT foci of southern Cameroon*.* The identification of *Wolbachia* with only one marker underestimates its infection rates and the combination of several markers enables achieving higher accuracy. Co-infections of *Wolbachia* and trypanosomes are not common and no association between these two microorganisms was revealed in *G. p. palpalis*. The tripartite association between tsetse fly, *Wolbachia* and trypanosomes seems to vary according to tsetse infested areas and a better understanding of this association may require additional studies aiming to evaluate the genetic polymorphism of *Wolbachia* as well as its density in tsetse flies.

## Abbreviations

AAT: Animal African trypanosomiasis
HAT: Human African trypanosomiasis
rDNA: Ribosomal DNA
WSP: *Wolbachia* surface protein
